# BRCA1 and BRCA2 in breast cancer families from Wales: moderate mutation frequency and two recurrent mutations in BRCA1.

**DOI:** 10.1038/bjc.1998.701

**Published:** 1998-12

**Authors:** J. M. Lancaster, M. E. Carney, J. Gray, J. Myring, C. Gumbs, J. Sampson, D. Wheeler, E. France, R. Wiseman, P. Harper, P. A. Futreal

**Affiliations:** Department of Surgery, Duke University Medical Center, Durham, NC 27710, USA.

## Abstract

Mutations in the BRCA1/BRCA2 genes account for varying proportions of breast cancer families studied, and demonstrate considerable variation in mutational spectra coincident with ethnic and geographical diversity. We have screened for mutations in 17 families from Wales with two or more cases of breast cancer under age 50 and/or ovarian cancer. Eight out of 17 (47%) families had demonstrable mutations. Six out of 17 (35%) carried BRCA1 mutations and 2 out of 17 (12%) carried BRCA2 mutations. Two recurrent mutations in BRCA1 were identified, which appear to represent founder mutations in this population. These data support the existence of additional breast and ovarian cancer susceptibility genes.


British Journal of Cancer (1998) 78(11), 1417–1420
© 1998 Cancer Research Campaign

### *BRCA1* and *BRCA2* in breast cancer families from Wales: moderate mutation frequency and two recurrent mutations in *BRCA1*

**JM Lancaster[1,2], ME Carney[2], J Gray[3], J Myring[3], C Gumbs[1], J Sampson[3], D Wheeler[3], E France[3], R Wiseman[4], P Harper[3] and PA Futreal[1,2,5]**

[1]Department of Surgery, [2]Division of Gynecologic Oncology, Duke University Medical Center, Durham, NC 27710, USA; [3]Institute of Medical Genetics, University Hospital of Wales, Heath Park, Cardiff C4F 4XN, UK; [4]Laboratory of Molecular Carcinogenesis, NIEHS, Research Triangle Park, Durham, NC 27709, USA; [5]Department of Genetics, Duke University Medical Center, Durham, NC 27710, USA

**Summary** Mutations in the *BRCA1/BRCA2* genes account for varying proportions of breast cancer families studied, and demonstrate considerable variation in mutational spectra coincident with ethnic and geographical diversity. We have screened for mutations in 17 families from Wales with two or more cases of breast cancer under age 50 and/or ovarian cancer. Eight out of 17 (47%) families had demonstrable mutations. Six out of 17 (35%) carried *BRCA1* mutations and 2 out of 17 (12%) carried *BRCA2* mutations. Two recurrent mutations in *BRCA1* were identified, which appear to represent founder mutations in this population. These data support the existence of additional breast and ovarian cancer susceptibility genes.

**Keywords:** *BRCA1*; *BRCA2*; breast and ovarian cancer; Wales

Two genes, *BRCA1* and *BRCA2*, have been identified as being causative in familial breast/ovarian cancer (Miki et al, 1994; Wooster et al, 1995). Carriers of germline mutations in these genes are at increased risk of developing breast and ovarian cancers. Analysis of *BRCA1/BRCA2* in high-risk families has demonstrated considerable heterogeneity in inactivating mutations, with a lower proportion of families attributable to mutations in either gene than had previously been predicted by statistical analyses (Castilla et al, 1994; Phelan et al, 1996; Serova et al, 1997). We have screened affected individuals from 17 high-risk Welsh breast and breast/ovarian cancer families for mutations in both *BRCA1* and *BRCA2* to determine the proportion of families attributable to defects in these genes. Recent experience with recurrent *BRCA1* and *BRCA2* mutations in the Ashkenazi Jewish and Icelandic populations (Neuhausen et al, 1996*a*; Offit et al, 1996; Thorlacius et al, 1996), coupled with studies of Welsh cystic fibrosis patients (Ashley, 1986; Cheadle et al, 1993; Lucotte and Hazout, 1995), suggests that screening for common genetic alterations in patients with familial breast and ovarian cancer in the Welsh population may prove a productive approach to the study of susceptibility in this group.

### PATIENTS AND METHODS

Seventeen families with two or more individuals diagnosed with breast cancer before age 50 years and/or ovarian cancer were selected under informed consent from patients attending the Department of Medical Genetics at the University Hospital of Wales, Cardiff, UK. All families were resident in Wales, with all obviously non-UK names excluded. Pedigrees were obtained by a registered nurse in a face-to-face interview with the patient. Verification of diagnosis was carried out by patient chart and pathology report review wherever possible. All families fulfilling inclusion criteria, who agreed to participate, were reported in the study. All coding exons for *BRCA1* and *BRCA2* were analysed by single-stranded conformation analysis (SSCA) in fragments of 200–300 bp (Miki et al, 1994; Lancaster et al, 1996*a*). Genomic DNA was amplified by the polymerase chain reaction (PCR) in a Perkin-Elmer 9600 Thermocycler (Perkin-Elmer, CA, USA). Reaction products were then electrophoresed on 0.5 × MDE (FMC, ME, USA) gels, dried and autoradiographed. Additionally, *BRCA1* exon 11 and *BRCA2* exons 10, 11 and 27 were screened for frameshift and nonsense mutations (producing premature stop codons) using the protein truncation test (PTT) (Lancaster et al, 1996*a*, 1996*b*). Exon 11 (*BRCA1*) and exons 10 and 27 (*BRCA2*) were amplified by PCR in one fragment, and exon 11 (*BRCA2*) in two fragments, in the presence of forward primers containing a T7 promoter and transcription/translation initiation sequence (primer sequences available from jmlanc@acpub.duke.edu). The PCR product was then subjected to an in vitro transcription/translation reaction (Promega, Madison, WI, USA), electrophoresed on a 10–20% sodium dodecyl sulphate (SDS)-polyacrylamide Ready-Gel (Biorad, Malvern, PA, USA), fixed, dried and autoradiographed. Sequencing templates were produced for samples showing aberrant mobility on SSCA or PTT and sequenced using a PRISM DyeDeoxy Terminator Cycle Sequencing Kit and an ABI PRIZM 377 Automated Fluorescent Sequencer (Applied BioSystems, CA, USA), according to manufacturer's instructions. Genotyping was carried out using fluorescent dye-labelled primer pairs analysed on the ABI377 with the Genescan software package. Sequences of primers are available from the Genome

*Received 31 October 1997*
*Revised 11 May 1998*
*Accepted 13 May 1998*

*Correspondence to:* PA Futreal, Box 2611, Duke University Medical Center, Durham, NC 27707, USA

**Table 1** Families screened for *BRCA1* and *BRCA2* mutations

| Family | Mutation | Effect | BrCa cases (*n*) | BrCa < 50 years cases (*n*) | OvCa cases (*n*) | Comments/other cancers |
|---|---|---|---|---|---|---|
| Families positive for *BRCA1* mutations | | | | | | |
| CDF2 | 4184delTCAA | ter1364 | 2 | 2 | 0 | |
| CDF7 | 4184delTCAA | ter1364 | 4 | 4 | 0 | Two bilateral BrCa cases |
| CDF10 | 2011insT | ter615 | 3 | 3 | 0 | One bilateral BrCa case 'abdominal' cancer |
| CDF13 | 2594delC | ter845 | 1 | 1 | 3 | |
| CDF128 | 2594delC | ter845 | 6 | 4 | 0 | Uterus, testicle, lung |
| CDF209 | 1997del4 | ter610 | 2 | 2 | 0 | |
| Families positive for *BRCA2* mutations | | | | | | |
| CDF107 | 4075delGT | ter1284 | 3 | 2 | 0 | BrCa maternal and paternal sides, Pancreas, prostate, cervix |
| CDF325 | 6287del4 | ter2047 | 4 | 2 | 0 | |
| Families screened negative for mutations | | | | | | |
| CDF1 | | | 4 | 4 | 0 | One bilateral BrCa case |
| CDF5 | | | 4 | 3 | 1 | One dual primary BrCa/OvCa |
| CDF8 | | | 4 | 2 | 0 | |
| CDF11 | | | 5 | 2 | 0 | |
| CDF12 | | | 1 | 1 | 3 | All affected < 40 years |
| CDF16 | | | 1 | 1 | 1 | OvCa age 48 years |
| CDF65 | | | 7 | 4 | 0 | |
| CDF85 | | | 4 | 2 | 2 | Cancer cervix, stomach (40s) |
| CDF312 | | | 4 | 2 | 0 | Cancer larynx |

ter, termination codon; BrCa, breast cancer; OvCa, ovarian cancer; del, deletion. Readers should refer to the Breast Cancer Information Core (BIC) for full, up-to-date mutation spectra in both genes.

Data Base (http:gdbwww.gdb.org/gdb/gdbtop.html). A database of updated *BRCA1* and *BRCA2* mutations is catalogued by the Breast Cancer Information Core Database at the BIC web site (http://www.nchgr.nih.gov/dir/lab_transfer/bic/).

### RESULTS

Inactivating mutations were identified in 8 out of 17 (47%) families – six in *BRCA1* (35%) and two in *BRCA2* (12%) (Table 1). All mutations produced frameshifts that were predicted to result in premature termination of protein synthesis. The average age at diagnosis of breast cancer in eight families carrying mutations was 40.7 years, compared with an average age of 48.6 years in nine families without mutations (not statistically significant). Seven out of eight (87%) families carrying mutations contained a very early onset case of breast cancer (< 40 years), compared with four out of nine (44%) families without mutations. Of 13 site-specific breast cancer families (no ovarian cancer), five (38%) contained *BRCA1* mutations and two (15%) *BRCA2* mutations. Of the five breast/ovarian families in the study, only one was found to be segregating a *BRCA1* mutation. No *BRCA2* mutations were observed in breast/ovarian families. Two recurrent *BRCA1* mutations were found, 4184del4 and 2594delC. DNA samples from the recurrent mutation carriers were genotyped for a series of polymorphic microsatellite markers both in the *BRCA1* gene (D17S855, D17S1322) and flanking (D17S1185, D17S1320, D17S1325) the *BRCA1* gene. Genotyping results for the recurrent mutation chromosomes are given in Table 2 and discussed below.

### DISCUSSION

In this study, 17 Welsh families with apparent hereditary breast/ovarian cancer were studied for *BRCA1/BRCA2* mutations.

**Table 2** Haplotypes of common *BRCA1* mutations in Wales

| Marker | Family (mutation) CDF2 (4184del4 – *BRCA1*) | CDF7 (4184del4 – *BRCA1*) | CDF13 (2594delC – *BRCA1*) | CDF128 (2594delC – *BRCA1*) |
|---|---|---|---|---|
| D17S1185 | ND | ND | 214 | 214 |
| D17S1320 | 170 | 170 | 172/176 | 172/176 |
| D17S855 | 157 | 157 | 149 | 149 |
| D17S1322 | 121 | 121 | 121/127 | 121/127 |
| D17S1325 | 196 | 196 | 214 | 214 |

Alleles given in basepairs. Markers are in centromeric to telomeric order. D17S855 and D17S1322 are both intragenic to *BRCA1*. ND, not done.

The results show that, overall, only approximately half of these families were found to be segregating either a *BRCA1* or *BRCA2* mutation. This proportion, although lower than that predicted by statistical analysis of a cohort of families collected for the linkage analysis, is in agreement with that observed in several other studies. Interestingly, recurrent mutations were observed in half of the families with mutations, all of whom appear to be unrelated. Families CDF2 and CDF7 were found to have a *BRCA1* 4184del4 mutation, whereas families CDF13 and CDF128 were found to have a *BRCA1* 2594delC mutation. DNA samples from the recurrent mutation carriers were genotyped for a series of polymorphic microsatellite markers in and flanking *BRCA1* to assess haplotype sharing which would be indicative of common founder effects for these two mutations (Table 2). A common 157-bp allele at the D17S855 intragenic marker was found in the 4184del4 families. This D17S855 allele was not detected in typing 94 chromosomes

© Cancer Research Campaign 1998

from unaffected Welsh control subjects and appears to be quite rare in this population (unpublished results). Other studies have also reported a D17S855 157-bp allele in association with the 4184del4 mutation (Neuhausen et al, 1996). However, in those families, a 118-bp allele at the adjacent intragenic marker D17S1322 has been reported, whereas CDF2 and CDF7 share a 121-bp D17S1322 allele in disequilibrium with the D17S855 allele. The 4184del4 mutation has been reported 14 times in individuals of various ethnic backgrounds, and appears to be segregating on at least two different haplotypes to which our data from Wales adds a third (Neuhausen et al, 1996*b*; Szabo and King, 1997). Two other families, CDF13 and CDF128, were found to be carrying a common 2594delC *BRCA1* mutation. Genotyping revealed a common haplotype for this mutation as well. An additional marker centromeric to *BRCA1*, D17S1185, was also typed to confirm allele sharing proximal to the ambiguous genotype at D17S1320. This mutation has also been reported frequently in Sweden and Denmark (Johannsson et al, 1996; Hakansson et al, 1997), with those families reported to share a common allele of D17S855, and has also been reported once in a family from the south of England (Campbell et al, 1997). As is the case for the *BRCA1* 4184del4 mutation, data for the *BRCA1* 2594delC mutation indicates that it is also probably a recurrent founder mutation in the Welsh population. Full genealogical tracing is ongoing for all families with recurrent mutations. Additionally, we hope to obtain DNA from other members of family CDF128 to assess *BRCA1* mutation status in individuals affected with uterine, lung and testicular carcinoma. This study suggests that screening for the two recurrent *BRCA1* mutations described here may be a useful first step in assessing Welsh hereditary breast/ovarian cancer families.

The mutation detection rate in this cohort compares favourably with families ascertained in other studies (Phelan et al, 1996; Szabo and King, 1997). The proportion of breast cancer families attributable to *BRCA1* and *BRCA2* mutations in this cohort is roughly similar to that described elsewhere in Britain (Szabo and King, 1997). However, the finding of only one of five breast/ovarian families with *BRCA1* mutations is less than has been reported elsewhere (Narod et al, 1995). Additionally, we failed to detect any instances of the 6503delTT mutation in *BRCA2* that has been seen frequently in British families (Mazoyer et al, 1996). These data support the observation that although the overall contribution of *BRCA1/BRCA2* to familial breast cancer may be the same among study groups, the distribution of mutations is quite variable with respect to ethnogeographical differences. *BRCA1* mutation detection sensitivity using SSCA alone is approximately 80% (Lancaster et al, 1997), and our detection rate in this study is probably increased by the additional use of the protein truncation test (PTT). However, even if SSCA and PTT together fail to identify 15% of mutations, our data still provide support for the view that a significant proportion of familial breast/ovarian cancer (approximately 35–53%) is not due to mutations in either *BRCA1* or *BRCA2*, and that other dominant breast cancer susceptibility genes may exist. Linkage analysis of families negative for *BRCA1* and *BRCA2* mutations would further support this philosophy, and we are currently collecting samples from other family members wherever possible. The finding of *BRCA1* mutations in only one out of five breast/ovarian cancer families suggests additional familial ovarian cancer gene(s) also exist. Patients tested for *BRCA1* and *BRCA2* mutations and found to be negative should, thus, be informed of the potential of mutations in other as yet unidentified breast and ovarian cancer susceptibility genes contributing to continued increased cancer risk.

### ACKNOWLEDGEMENTS

We would like to thank Shazia Ali for outstanding technical support. This work was supported in part by the NCI/Duke University SPORE (Specialised Program of Research Excellence) in breast cancer (P50-CA68348) and the TRACE (Trial of Genetic Assessment in breast cancer) project funded by the Medical Research Council and the Welsh Office. L.F. is funded by the Imperial Cancer Research Fund. J.G. is supported by NHS (Wales) Research and Development.

### REFERENCES

Ashley DJB (1986) Welshness and disease. In *Genetic and Population Studies in Wales*. Harper PS (ed.). University of Wales Press: Cardiff

Campbell IG, Schroff R, Englefield P and Eccles DM (1997) *BRCA1* polymorphisms. *Br J Cancer* **75**: 1854–1855

Castilla LH, Couch FJ, Erdos MR, Hoskins KF, Calzone K, Garber JE, Boyd J, Lubin MB, Deshano ML, Brody LC, Collins FS and Weber BL (1994) Mutations in the *BRCA1* gene in families with early-onset breast and ovarian cancer. *Nature Genet* **8**: 387–391

Cheadle JP, Goodchild MC and Meredith AL (1993) Direct sequencing of the complete CFTR gene: the molecular characterisation of 99.5% of CF chromosomes in Wales. *Hum Mol Genet* **2**: 1551–1556

Hakansson S, Johannsson O, Johansson U, Sellberg G, Loman N, Gerdes AM, Holmberg E, Dahl N, Pandis N, Kristoffersson U, Olsson H and Borg A (1997) Moderate frequency of *BRCA1* and *BRCA2* germ-line mutations in Scandinavian familial breast cancer. *Am J Hum Genet* **60**: 1068–1078

Johannsson O, Ostermeyer EA, Hakansson S, Friedman LS, Johansson U, Sellberg G, Brondum-Nielsen K, Sele V, Olsson H, King MC and Borg A (1996) Founding *BRCA1* mutations in hereditary breast and ovarian cancer in southern Sweden. *Am J Hum Genet* **58**: 441–450

Lancaster JM, Wooster R, Mangion J, Phelan CM, Cochran C, Gumbs C, Seal S, Barfoot R, Collins N, Bignell G, Patel S, Hamoudi R, Larsson C, Wiseman R, Berchuck A, Iglehart JD, Marks JR, Ashworth A, Stratton M and Futreal PA (1996*a*) *BRCA2* mutations in primary breast and ovarian cancers. *Nature Genet* **13**: 238–240

Lancaster JM, Cochran CJ, Browlee HA, Berchuck A, Futreal PA and Wiseman RW (1996*b*) Detection of *BRCA1* mutations in women with early-onset ovarian cancer by use of the protein truncation test. *J Natl Cancer Inst* **88**: 552–554

Lancaster JM, Futreal A, Berchuck A and Wiseman RW (1997) The dideoxy fingerprinting assay for *BRCA1* mutation analysis. *Mol Carcinog* **19**: 176–179

Lucotte G and Hazout S (1995) Geographic and ethnic distributions of the more frequent cystic fibrosis mutations in Europe show that a founder effect is apparent for several mutant alleles. *Hum Biol* **67**: 562–576

Mazoyer S, Dunning AM, Serova O, Dearden J, Puget N, Healey CS, Gayther SA, Mangion J, Stratton MR, Lynch HT, Goldgar DE, Ponder BA and Lenoir GM (1996) A polymorphic stop codon in *BRCA2*. *Nature Genet* **13**: 245–247

Miki Y, Swensen J, Shattuck-Eidens D, Futreal PA, Harshman K, Tavtigian S, Liu Q, Cochran C, Bennett LM, Ding W, Bell R, Rosenthal J, Hussey C, Tran T, McClure M, Frye C, Hattier T, Phelps R, Haugen-Strano A, Katcher H, Yakumo K, Gholami Z, Shaffer D, Stone S, Bayer S, Wray C, Borgden R, Dayananth P, Ward J, Tonin P, Narod S, Bristow P, Norris F, Helvering L, Morrision P, Roseteck P, Lai M, Barrett JC, Lewis C, Neuhausen S, Cannon-Albright L, Goldgar D, Wiseman R, Kamb A and Skolnick MH (1994) A strong candidate for the breast and ovarian cancer susceptibility gene *BRCA1*. *Science* **266**: 66–71

Narod SA, Ford D, Devilee P, Barkardottir RB, Lynch HT, Smith SA, Ponder BA, Weber BL, Garber JE, Birch JM and the Breast Cancer Linkage Consortium (1995) An evaluation of genetic heterogeneity in 145 breast-ovarian cancer families. *Am J Hum Genet* **56**: 254–264

Neuhausen S, Gilewski T, Norton L, Tran T, McGuire P, Swensen J, Hampel H, Borgen P, Brown K, Skolnick M, Shattuck-Eidens D, Jhanwar S, Goldgar D and Offit K (1996*a*) Recurrent *BRCA2* 6174delT mutations in Ashkenazi Jewish women affected by breast cancer. *Nature Genet* **13**: 126–128

Neuhausen SL, Mazoyer S, Friedman L, Stratton M, Offit K, Caligo A, Tomlinson G, Cannon-Albright L, Bishop T, Kelsell D, Solomon E, Weber B, Couch F, Struewing J, Tonin P, Durocher F, Narod S, Skolnick MH, Lenoir G, Serova O, Ponder B, Stoppa-Lyonnet D, Easton D, King MC and Goldgar DE (1996*b*) Haplotype and phenotype analysis of six recurrent *BRCA1* mutations in 61 families: results of an international study. *Am J Hum Genet* **58**: 271–280

© Cancer Research Campaign 1998

Offit K, Gilewski T, McGuire P, Schluger A, Hampel H, Brown K, Swensen J, Neuhausen S, Skolnick M, Norton L and Goldgar D (1996) Germline *BRCA1* 185delAG mutations in Jewish women with breast cancer. *Lancet* **347**: 1643–1645

Phelan CM, Lancaster JM, Tonin P, Gumbs C, Cochran C, Carter R, Ghadirian P, Perret C, Faucher MC, Dole K, Karimi S, Foulkes W, Lounis H, Warner E, Gross P, Anderson D, Larsson C, Narod SA and Futreal PA (1996) Mutation analysis of the *BRCA2* gene in 49 site-specific breast cancer families. *Nature Genet* **13**: 120–122

Serova OM, Mazoyer S, Puget N, Dubois V, Tonin P, Shugart YY, Goldgar D, Narod SA, Lynch HT and Lenoir GM (1997) Mutations in *BRCA1* and *BRCA2* in breast cancer families: are there more breast cancer-susceptibility genes? *Am J Hum Genet* **60**: 486–495

Szabo C and King MC (1997) Population genetics of *BRCA1* and *BRCA2*. *Am J Hum Genet* **60**: 1013–1020

Thorlacius S, Olafsdottir G, Tryggvadottir L, Neuhausen S, Jonasson JG, Tavtigian SV, Tulinius H, Ogmundsdottir HM and Eyfjord JE (1996) A single *BRCA2* mutation in male and female breast cancer families from Iceland with varied cancer phenotypes. *Nature Genet* **13**: 117–119

Wooster R, Bignell G, Lancaster J, Swift S, Seal S, Mangion J, Collins N, Gumbs C, Mickle G, Barfoot R, Hamoudi R, Patel S, Rice C, Biggs P, Hashim Y, Smith A, Connor F, Arason A, Gudmundsson J, Ficenec D, Kelsell D, Ford D, Tonin P, Bishop DT, Spurr NK, Ponder BAJ, Ellese R, Peto J, Devilee P, Cornelisse C, Lynch H, Narod S, Lenor G, Egilsson V, Barkadottir RB, Eason DF, Bentley DR, Futreal PA, Ashworth A and Stratton MR (1995) Identification of the breast cancer susceptibility gene *BRCA2*. *Nature* **378**: 789–792

*© Cancer Research Campaign 1998*


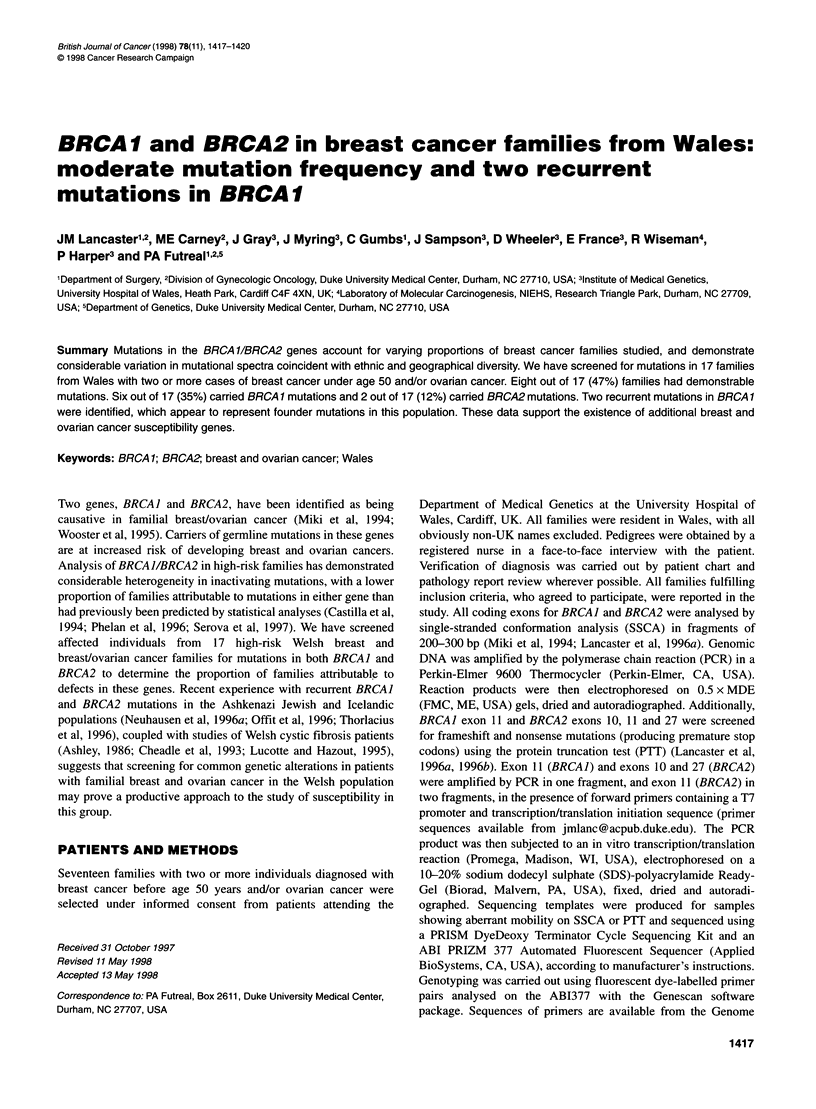

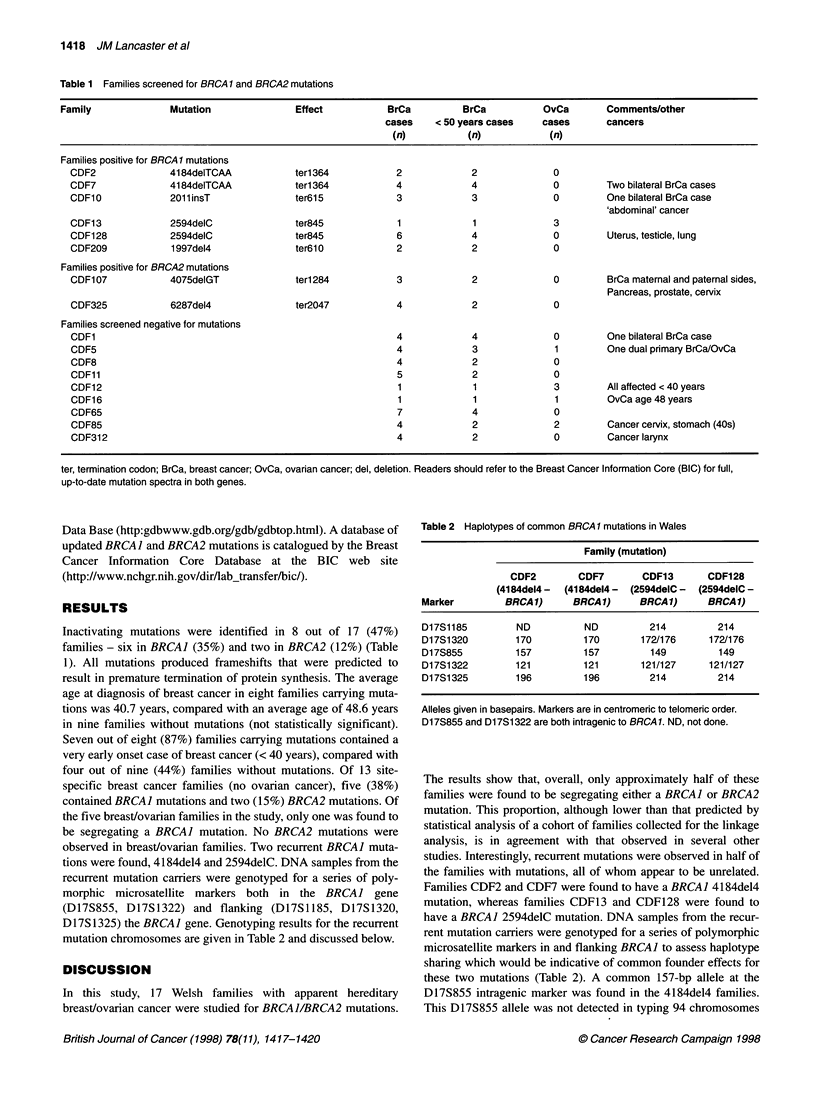

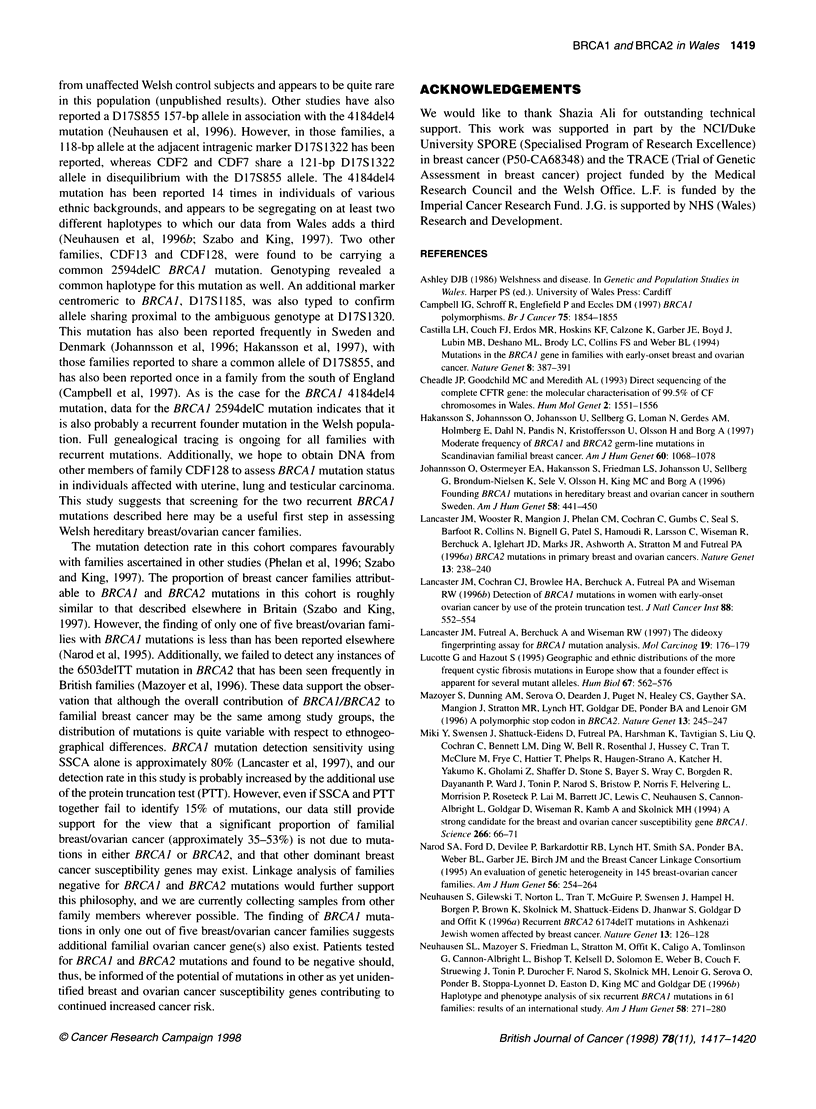

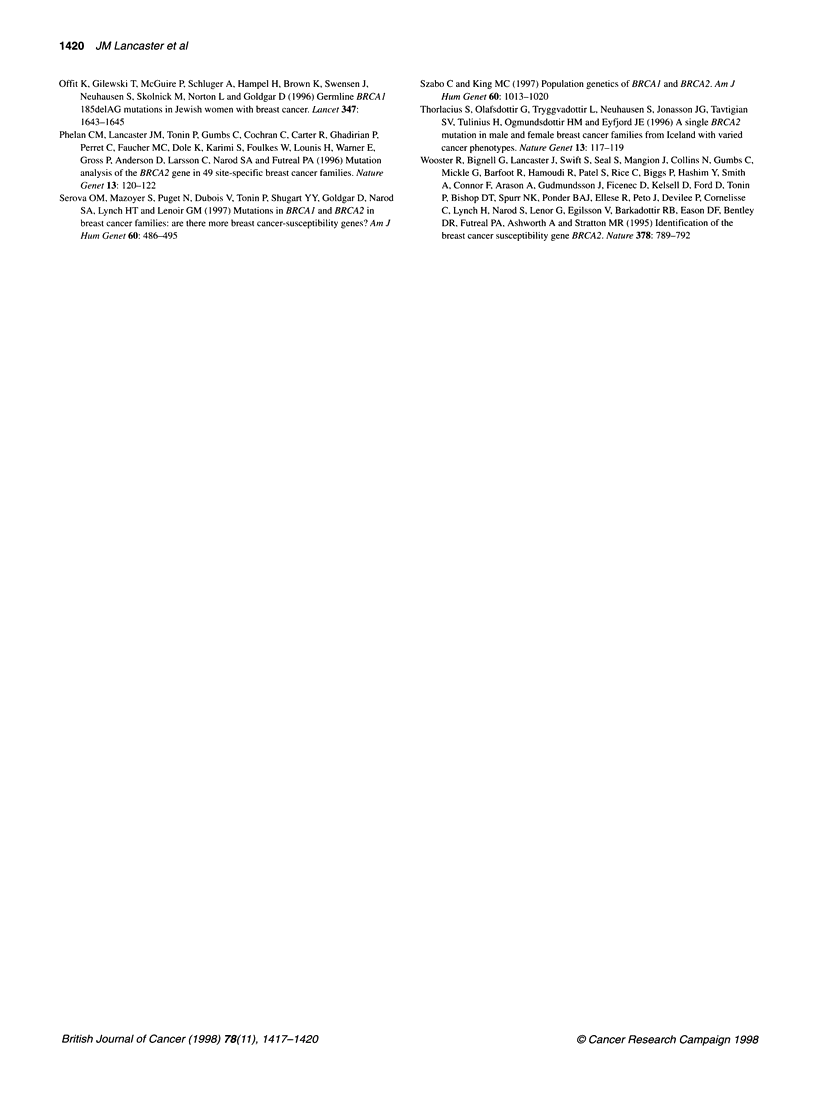


## References

[OCR_00303] Campbell I. G., Schroff R., Englefield P., Eccles D. M. (1997). BRCA1 polymorphisms.. Br J Cancer.

[OCR_00307] Castilla L. H., Couch F. J., Erdos M. R., Hoskins K. F., Calzone K., Garber J. E., Boyd J., Lubin M. B., Deshano M. L., Brody L. C. (1994). Mutations in the BRCA1 gene in families with early-onset breast and ovarian cancer.. Nat Genet.

[OCR_00314] Cheadle J. P., Goodchild M. C., Meredith A. L. (1993). Direct sequencing of the complete CFTR gene: the molecular characterisation of 99.5% of CF chromosomes in Wales.. Hum Mol Genet.

[OCR_00319] Håkansson S., Johannsson O., Johansson U., Sellberg G., Loman N., Gerdes A. M., Holmberg E., Dahl N., Pandis N., Kristoffersson U. (1997). Moderate frequency of BRCA1 and BRCA2 germ-line mutations in Scandinavian familial breast cancer.. Am J Hum Genet.

[OCR_00326] Johannsson O., Ostermeyer E. A., Håkansson S., Friedman L. S., Johansson U., Sellberg G., Brøndum-Nielsen K., Sele V., Olsson H., King M. C. (1996). Founding BRCA1 mutations in hereditary breast and ovarian cancer in southern Sweden.. Am J Hum Genet.

[OCR_00348] Lancaster J. M., Berchuck A., Futreal P. A., Wiseman R. W. (1997). Dideoxy fingerprinting assay for BRCA1 mutation analysis.. Mol Carcinog.

[OCR_00341] Lancaster J. M., Cochran C. J., Brownlee H. A., Evans A. C., Berchuck A., Futreal P. A., Wiseman R. W. (1996). Detection of BRCA1 mutations in women with early-onset ovarian cancer by use of the protein truncation test.. J Natl Cancer Inst.

[OCR_00335] Lancaster J. M., Wooster R., Mangion J., Phelan C. M., Cochran C., Gumbs C., Seal S., Barfoot R., Collins N., Bignell G. (1996). BRCA2 mutations in primary breast and ovarian cancers.. Nat Genet.

[OCR_00351] Lucotte G., Hazout S. (1995). Geographic and ethnic distributions of the more frequent cystic fibrosis mutations in Europe show that a founder effect is apparent for several mutant alleles.. Hum Biol.

[OCR_00375] Narod S. A., Ford D., Devilee P., Barkardottir R. B., Lynch H. T., Smith S. A., Ponder B. A., Weber B. L., Garber J. E., Birch J. M. (1995). An evaluation of genetic heterogeneity in 145 breast-ovarian cancer families. Breast Cancer Linkage Consortium.. Am J Hum Genet.

[OCR_00387] Neuhausen S. L., Mazoyer S., Friedman L., Stratton M., Offit K., Caligo A., Tomlinson G., Cannon-Albright L., Bishop T., Kelsell D. (1996). Haplotype and phenotype analysis of six recurrent BRCA1 mutations in 61 families: results of an international study.. Am J Hum Genet.

[OCR_00384] Neuhausen S., Gilewski T., Norton L., Tran T., McGuire P., Swensen J., Hampel H., Borgen P., Brown K., Skolnick M. (1996). Recurrent BRCA2 6174delT mutations in Ashkenazi Jewish women affected by breast cancer.. Nat Genet.

[OCR_00402] Offit K., Gilewski T., McGuire P., Schluger A., Hampel H., Brown K., Swensen J., Neuhausen S., Skolnick M., Norton L. (1996). Germline BRCA1 185delAG mutations in Jewish women with breast cancer.. Lancet.

[OCR_00414] Serova O. M., Mazoyer S., Puget N., Dubois V., Tonin P., Shugart Y. Y., Goldgar D., Narod S. A., Lynch H. T., Lenoir G. M. (1997). Mutations in BRCA1 and BRCA2 in breast cancer families: are there more breast cancer-susceptibility genes?. Am J Hum Genet.

[OCR_00423] Szabo C. I., King M. C. (1997). Population genetics of BRCA1 and BRCA2.. Am J Hum Genet.

[OCR_00427] Thorlacius S., Olafsdottir G., Tryggvadottir L., Neuhausen S., Jonasson J. G., Tavtigian S. V., Tulinius H., Ogmundsdottir H. M., Eyfjörd J. E. (1996). A single BRCA2 mutation in male and female breast cancer families from Iceland with varied cancer phenotypes.. Nat Genet.

[OCR_00433] Wooster R., Bignell G., Lancaster J., Swift S., Seal S., Mangion J., Collins N., Gregory S., Gumbs C., Micklem G. (1995). Identification of the breast cancer susceptibility gene BRCA2.. Nature.

